# Context-specific functional module based drug efficacy prediction

**DOI:** 10.1186/s12859-016-1078-6

**Published:** 2016-07-28

**Authors:** Woochang Hwang, Jaejoon Choi, Mijin Kwon, Doheon Lee

**Affiliations:** 1 Department of Bio and Brain Engineering, 291 Daehak-ro, Yuseong-gu, Daejeon, Republic of Korea; 2 Bio-Synergy Research Center, 291 Daehak-ro, Yuseong-gu, Daejeon, Republic of Korea

## Abstract

**Background:**

It is necessary to evaluate the efficacy of individual drugs on patients to realize personalized medicine. Testing drugs on patients in clinical trial is the only way to evaluate the efficacy of drugs. The approach is labour intensive and requires overwhelming costs and a number of experiments. Therefore, preclinical model system has been intensively investigated for predicting the efficacy of drugs. Current computational drug sensitivity prediction approaches use general biological network modules as their prediction features. Therefore, they miss indirect effectors or the effects from tissue-specific interactions.

**Results:**

We developed cell line specific functional modules. Enriched scores of functional modules are utilized as cell line specific features to predict the efficacy of drugs. Cell line specific functional modules are clusters of genes, which have similar biological functions in cell line specific networks. We used linear regression for drug efficacy prediction. We assessed the prediction performance in leave-one-out cross-validation (LOOCV). Our method was compared with elastic net model, which is a popular model for drug efficacy prediction. In addition, we analysed drug sensitivity-associated functions of five drugs - lapatinib, erlotinib, raloxifene, tamoxifen and gefitinib- by our model.

**Conclusions:**

Our model can provide cell line specific drug efficacy prediction and also provide functions which are associated with drug sensitivity. Therefore, we could utilize drug sensitivity associated functions for drug repositioning or for suggesting secondary drugs for overcoming drug resistance.

**Electronic supplementary material:**

The online version of this article (doi:10.1186/s12859-016-1078-6) contains supplementary material, which is available to authorized users.

## Background

**Fig. 1:**
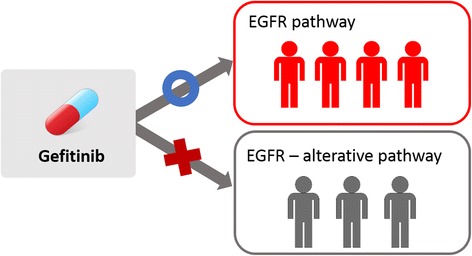
The difference of drug response. The difference of activated pathway can change the drug response

It is important to predict drug efficacy by genomic disease signatures for realizing personalized therapy. Although people have same disease, they show different status of genomic signatures, and it causes different efficacy of a drug. For example, Gefitinib is a first-line drug for advanced non-small-cell lung carcinoma (NSCLC) patients, but only 20 ~ 30 % patients are sensitive to Gefitinib (Fig. 1) [1].

There are two types of methods for identifying the efficacy of a drug; clinical trials and computational methods. Although clinical trial is much accurate in assessing drug efficacy and toxicity, it requires overwhelming cost and a number of tests. Also, there is a limitation in experimental method, for it cannot predict the efficacy of a new drug. So, we need to conduct same overall process of clinical trial to identify the efficacy of a new drug.

There are, accordingly, many computational methods which predict the efficacy of a new drug using genomic data [2, 3]. With the recent advances biological experimental technologies, large collections of matched drug screens and genomics profiles of cancer cell lines have been published [4, 5]. These data have been used to build drug efficacy prediction models by associating genomic features with drug sensitivity in cancer cell lines [6–9]. These previous studies used single gene or multi genes as associated genomic features for predicting drug efficacy.

In tumorigenesis, diverse patterns of mutation, gene expression have been observed in cancer-specific, or tissue - specific manner [10]. Diverse patterns of genomic features according to the biological contexts play an important role in clinical efficacy. Recently it has been found that biological networks can be rewired according to biological contexts, such as genotype and phenotype [11–14]. With network rewiring, drug responses in each person can be changed [15]. For example, in Gefitinib-sensitive cancers, RAS,MEK/ERK and PI3K/AKT signaling pathways are suppressed, resulting in cell cycle arrest and apoptosis. In Gefitinib-resistant cancers with network rewiring, the secondary RTK, which is not a target of Gefitinib, reactivates RAS,MEK/ERK and PI3K/AKT signaling pathways. Sustained activations of these pathways result in cell proliferation and survival in the presence of Gefitinib.

Previous methods used known gene sets or known pathways as their features for predicting drug efficacy. Therefore, those methods cannot consider network rewiring.

By considering network rewiring and biological context, we can enhance the accuracy in predicting drug efficacy. We assume that each cell line has differently activated gene set of same biological functions, so if activated gene sets of each cell line are similar, the drug efficacy of cell lines is similar. For instance, activated gene sets of apoptosis are similar in cell line1 and cell line 2. In this case, the efficacy of Lapatinib, a drug related to apoptosis, will be similar in both cell line 1 and cell line 2. To be generalized, this method comparing the functions of a drug and the functions associated to the activated gene sets in a cell line explains the efficacy and related biological functions of a drug.

Here, we aim to develop a method considering network rewiring and biological context to predict the efficacy of drugs. This method will suggest personalized medicines based on genomic information.

## Methods

**Fig. 2:**
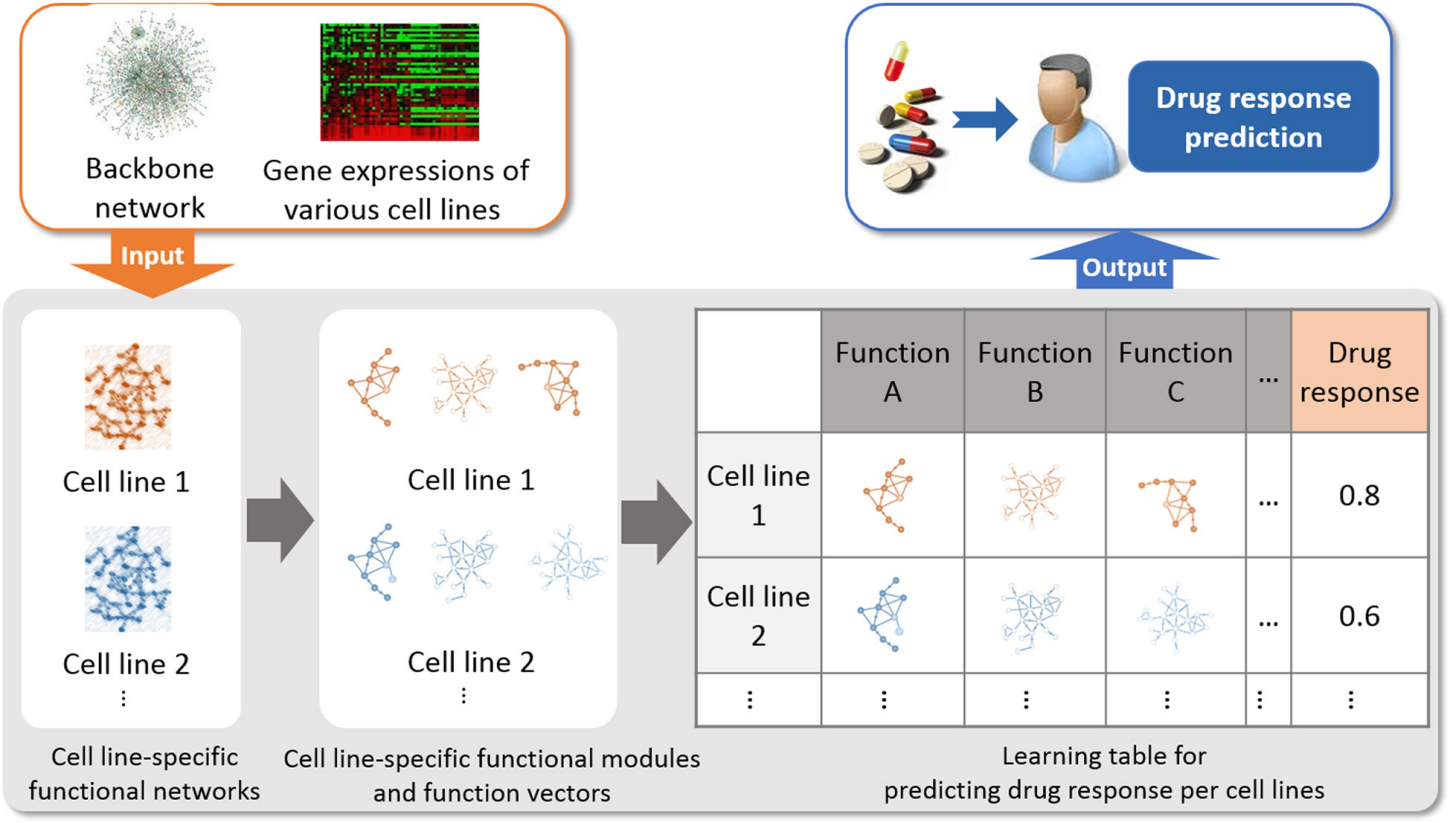
System overview. 1. We constructed the backbone network. 2. We made cell line-specific networks using gene expression data of cell lines from NCI60 and the backbone network. 3. We identified cell line-specific function modules applying network clustering algorithm on the cell line specific network. 4. We identified context-specific functions by calculating module similarity of each functional module. 5. We assigned cell line-specific functional modules on cell line-specific functions to make learning models for predicting the efficacy of drugs. 6. We predicted the efficacy of drugs

We explained system overview in Fig. 2.

### Data preprocessing

We used gene expression data of NCI-60 [16], a panel of 60 diverse human cancer cell lines. The gene expression data of 9 different cancer types is from GSE32474 and GSE34211 in GEO database. We normalized the gene expression data of cancer samples from each cell line, which passed quality check, by GCRMA. The gene expression data of 9 normal tissues are arranged from GSE21422, GSE15824, GSE8671, GSE48060, GSE30999, GSE11842, GSE14407, GSE55945, and E-TABM-282, respectively, in GEO and arrayexpress databases. We normalized the gene expression data of normal samples which passed quality check, by GCRMA.

### Biological network construction

We constructed a backbone network by integrating public databases, which are BioGrid [17], KEGG [18], and TRANFAC [19]. The constructed backbone network includes various types of interactions such as protein-protein interactions and gene regulatory interactions. The backbone network has 12,849 nodes and 300,507 interactions.

### Context-specific function module

We used MCL for clustering the backbone network. MCL is a graph clustering using flow simulation. Several researches utilized and proved that MCL generates robust cluster functional modules from given biological networks [20–23]. Through MCL, we could generate MCL functional modules of the backbone network. We assigned absolute value of PCC of the two genes connected in the network as edge weights. We used 2.5 as the inflation coefficient. For analysing the clustering result, we chose MCL modules of size greater than 8.

### Function vector

A function vector is a vector containing GO terms that are enriched on genes of a functional module. Each functional module has multiple enriched GO terms, which are biological functions. Therefore, it is difficult to identify the function of a functional module.

**Fig. 3:**
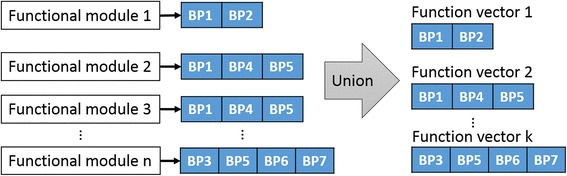
Function vector. Function vectors are vectors containing enriched GO terms of functional modules

To assign a function on a functional module, we made a function vector. To find all function vectors, we conducted following steps; first, we performed enrichment analysis to find enriched GO terms of all functional modules. We made enriched GO terms as a vector. Second, we eliminated repeated vectors of GO terms (Fig. 3).

### Context-specific function detection

We could obtain module similarities between GO terms of functional modules and function vectors by calculating Jaccard index:

$$ \begin{array}{l}J\left(FM,\kern0.5em FV\right)\\ {}=\frac{\left|FM\cap \kern0.5em FV\right|}{\left|FM\cup \kern0.5em FV\right|}\\ {}FM:\kern0.5em  Function\kern0.5em  module,\\ {}FV:\kern0.5em  Function\kern0.5em  vector\end{array} $$

**Fig. 4:**
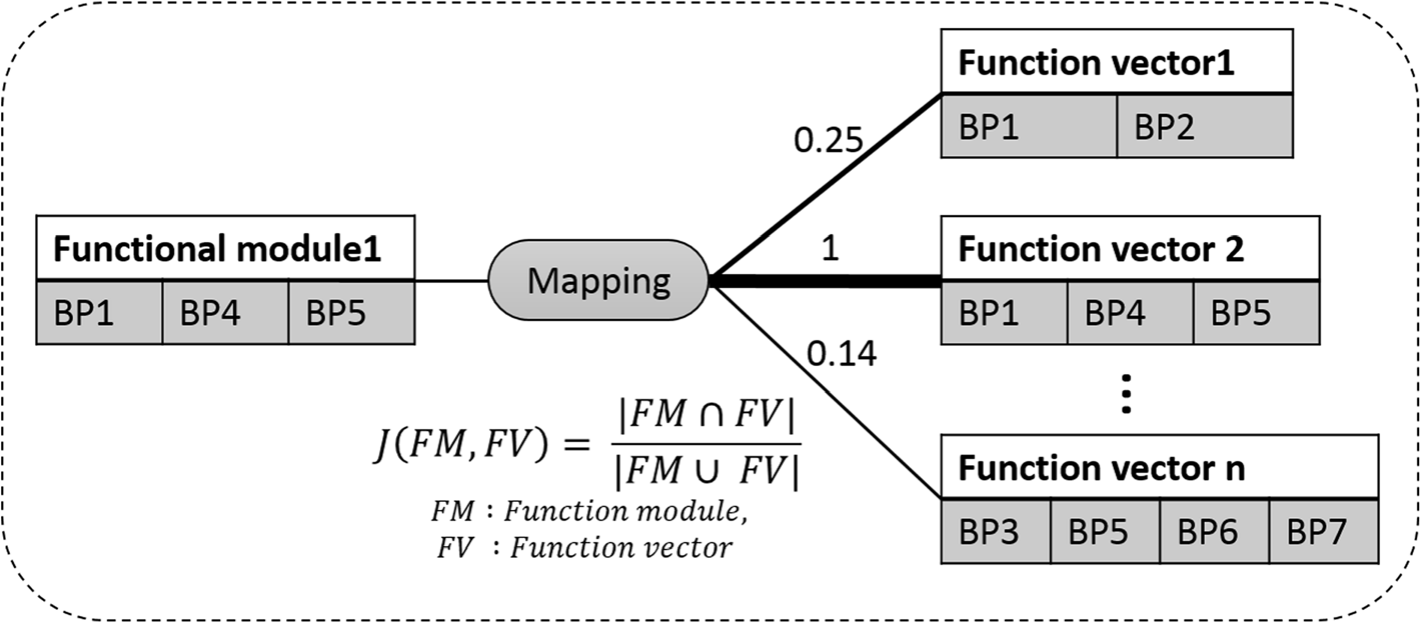
Mapping a function module to a function vector. Functional module 1 mapped on a function vector 2

We utilized module similarities between function modules and function vectors to map each function module on corresponding function vector in a learning table. First, we calculated module similarities between functional modules and whole function vectors. Second, we mapped functional modules on function vectors, which have the highest module similarity between GO terms of the functional module and function vectors (Fig. 4).

### Regression model

We made learning tables for performing a multiple linear regression per a drug. First, we found functional modules of cell lines. Second, we found function vectors. Third, we mapped each functional module of each cell line on function vectors. Forth, we calculated a functional module score by:

$$ \mathrm{Module}\kern0.5em \mathrm{score}=\frac{{\displaystyle \sum F{C}_i}}{N}, $$

where FCi represents fold change of gene i and N represents the number of genes in a module.

Fifth, we added GI50, which is drug concentration required to reduce growth rates to 50 % of the maximum rate, values of drugs as drug response values.

The multiple linear regression model is defined as the expected value of y conditioned on values of x:

$$ \mathrm{y}={\gamma}_1{x}_1+\cdots {\gamma}_n{x}_n+\varepsilon, $$

where y represents GI50 value of a drug and x_i_ represents score of a functional module i.

## Results and discussion

### Context-specific functional module

To make context-specific function modules, we first construct context-specific networks that mean cell line-specific networks in this work. We calculate the Pearson correlation coefficients (PCCs) for all interactions in the backbone network to construct context-specific networks. The criteria for context-specific interaction is greater than p-value 0.01 of PCCs. Then we assign values of PCCs as edge weights of context-specific network. Next, we use network clustering algorithm, MCL (Markov clustering) [20], to detect functional modules in the weighted context-specific network. MCL algorithm cluster weighted network by making strongly correlated edges to get stronger and making weakly correlated edges to get weaker. Thereby, only strongly correlated edges are survived.

The MCL clusters many network modules, the majority of which are very small, and contain two or three genes only. we filtered the modules by an arbitrary threshold n and selected *n* = 8 by reference [20].

### Context-specific function

Context-specific functional module has more than one enriched GO biological processes. Thereby, it is difficult to identify related function of a context-specific functional module. For example, “MCF Module 1”, which is one of context-specific functional modules, has three enriched GO biological processes, which are “GO: 1234”, “GO:156” and “GO:3249”. Enriched GO biological processes of “MCF Module 2” are “GO: 1234”, “GO: 145” and “GO: 3244”. A drug efficacy prediction model of Gefitinib suggests that “GO:1234” is associated with efficacy of Gefitinib. Then, we cannot identify whether related function of Gefitinib efficacy is “MCF Module 1” or “MCF Module 2”. To avoid this ambiguousness, we define a GO vector for mapping one context-specific function module to a function. The GO vector is called context-specific function vector in this work. In this example, the context-specific function vector of ‘MCF Module 1” is [“GO: 1234”, “GO: 156”, “GO: 3249”]. We assign context-specific function vector of context-specific functional module by module similarity method.

**Fig. 5:**
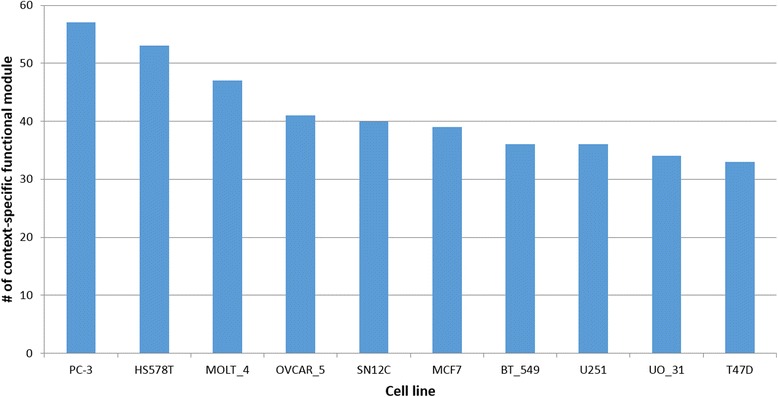
The number of condition specific functional modules of cell lines

We identified 715 context specific functional modules, and the number of modules in each cell line is shown in Fig. 5. The number of function modules of all cell lines is in Additional file 1. Using these context-specific functional modules, we identified 594 context-specific function vectors (Table 1).

**Table 1:** Context-specific function vectors

Function	GO ID	GO description
1	GO:0000377	RNA splicing, via transesterification reactions with bulged adenosine as nucleophile
	GO:0000375	RNA splicing, via transesterification reactions
	GO:0006139	Nucleobase, nucleoside, nucleotide and nucleic acid metabolic process
	GO:0006396	RNA processing
	GO:0000398	Nuclear mRNA splicing, via spliceosome
	GO:0016070	RNA metabolic process
	GO:0044260	Cellular macromolecule metabolic process
305	GO:0002520	Immune system development
	GO:0002329	Pre-B cell differentiation
	GO:0030097	Hemopoiesis
	GO:0048534	Hemopoietic or lymphoid organ development
	GO:0002327	Immature B cell differentiation
472	GO:0051056	Regulation of small GTPase mediated signal transduction
	GO:0050790	Regulation of catalytic activity
	GO:0043087	Regulation of GTPase activity

### Performance of drug efficacy prediction

**Fig. 6:**
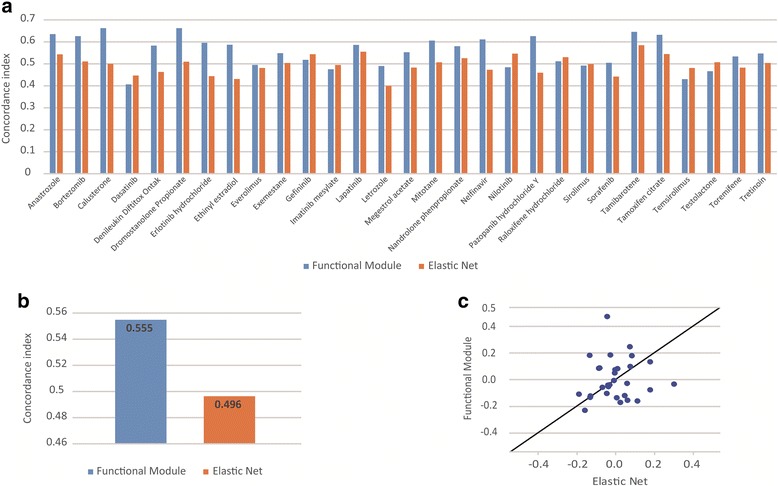
Performance comparison of our model with elastic net. **a** Prediction performance of leave-one-out cross-validation (LOOCV) in the NCI60, as quantified by the concordance index between the predicted and observed GI50 values. **b** Comparison of the average concordance index of 29 drugs. **c** Pearson correlation coefficients between the prediction and the observed data are calculated for each algorithm. The correlation coefficients from elastic net (x-axis) are compared to those from our model (y-axis). Each dot represents prediction performance for GI50 value of one drug

NCI60 has drug response data of more than 2000 drugs. We used drugs which are FDA approved and targeted therapy. Thereby, we predicted GI50 values of 29 drugs, which are tyrosine kinase inhibitors, hormones or interleukins. We then validated predictors’ performance by computing the concordance index, which is a generalization of the area under the receiving characteristics operating curve [3]. The concordance index estimates the probability of how correctly the model predicts which are the most and the least sensitive cell lines to a drug. A random predictor would be 0.5, while a perfect predictor would be 1. Value of c-index, which represents correctness of the predicted drug efficacy, is shown in Fig. 6a. We made a multiple linear regression model of each drug to predict efficacy of a drug. We used leave-one-out-cross-validation (LOOCV).

To validate context-specific functional modules are significant features for predicting the efficacy of drugs, we compared our model with elastic net, which is efficient, widely used regularized regression technique [5]. As can be seen in Fig. 6a, our model gives better predictive performance than elastic net for 21 out of 29 drugs and we observed a significant good performance for 11 out of 29 drugs (Anastrozole, Bortezomib, Calusterone, Dromostanolone Propionate, Erlotinib, Ethinyl estradiol, Mitotane, Nelfinavir, Pazopanib hydrochloride Y, Tamibarotene, Tamoxifen citrate) (Additional file 2) , our model yielding a concordance index greater than 0.586 (*p* < 0.05). Elastic net gives significant performance for one out of 29 drugs (Tamibarotene). To compare performance result, we applied one tail paid *t*-test for comparing concordance index with Person correlation [24]. Our method outperforms elastic net (*p* < 2e-4 one tail paired *t*-test, for comparing concordance index; *p* < 0.027 for comparing Person correlation) (Additional file 3). It is shown in Fig. 6c.

### Lapatinib, Erlotinib

In our predicted result, drug efficacy related context-specific function of Lapatinib are Function 305 and Function 66. Function 305 is related to immune system development and Function66 is related to regulation of JAK-STAT cascade and cell proliferation. Lapatinib blocks EGFR, which is a target of Lapatinib. So, it makes EGFR not to transfer signal to JAK-STAT pathway. Thereby, Lapatinib negatively regulates cell proliferation. Among the drugs we experimented, Erlotinib has same therapeutic function as Lapatinib. As we expected, context-specific functions which are related to efficacy of Erlotinib are the same with as Lapatinib.

### Raloxifene, Tamoxifen

Raloxifene targets estrogen receptor and it acts as estrogen agoinst [25]. In our research, the context-specific function of Raloxifene is Function 501 (Additional file 4). Function 501 is related to tissue morphogenesis [26]. One of the functions of estrogen is tissue morphogenesis [27]. Tamoxifen, which we experimented, has same therapeutic function as Raloxifene. The Function 501 is on top4 context-specific function of Tamoxifen [28].

### Gefitinib

GTPase activity is the context-specific function of Gefitinib in our experiment. In cellular environment, KRAS transfers signal to downstream pathways by GTPase activity. If KRAS has mutation, it consistently activates downstream pathways and causes resistance to Gefitinib [29].

## Conclusions

A clinical trial validates the efficacy of personalized medicines, but does not predict it. To develop personalized medicines, it is necessary to predict the efficacy of drugs using individual genomic information. Therefore, many groups have studied approaches to predict the efficacy of drugs, but they could not explain which biological functions are related to drug activity. The context-specific function module based approach not only predicts the efficacy of drugs but also describes drug-related biological functions. In this paper, we generated the model which predicts efficacy of drugs, using 60 cell lines from NCI 60. We expect that this model will show better performance if based on larger amount of cell line data from databases such as CCLE. The proposed approach predicts secondary drugs for resistant drugs as well as suggests personalized drugs.

## Additional files

Additional file 1: Number of functional modules (TIF 161 kb) Additional file 2: Comparison concordance index between our model and elastic net. It contains concordance index of our model and elastic net in each drug. It contains p-value of concordance index of our model and elastic net in each drug. It contains drug target information. (XLSX 11 kb) Additional file 3: Comparison Pearson correlation coefficient between our model and elastic net. It contains correlation coefficient of our model and elastic net in each drug. (XLSX 9 kb) Additional file 4: GO terms of function vectors. Number of context-specific functional modules of cell lines (DOCX 18 kb)
