# Supplementary figures and images for: Context-specific functional module based drug efficacy prediction
Source: BMC Bioinformatics. 2016 Jul 28;17(Suppl 6):275. doi: 10.1186/s12859-016-1078-6 (PMC4965733; doi:10.1186/s12859-016-1078-6)

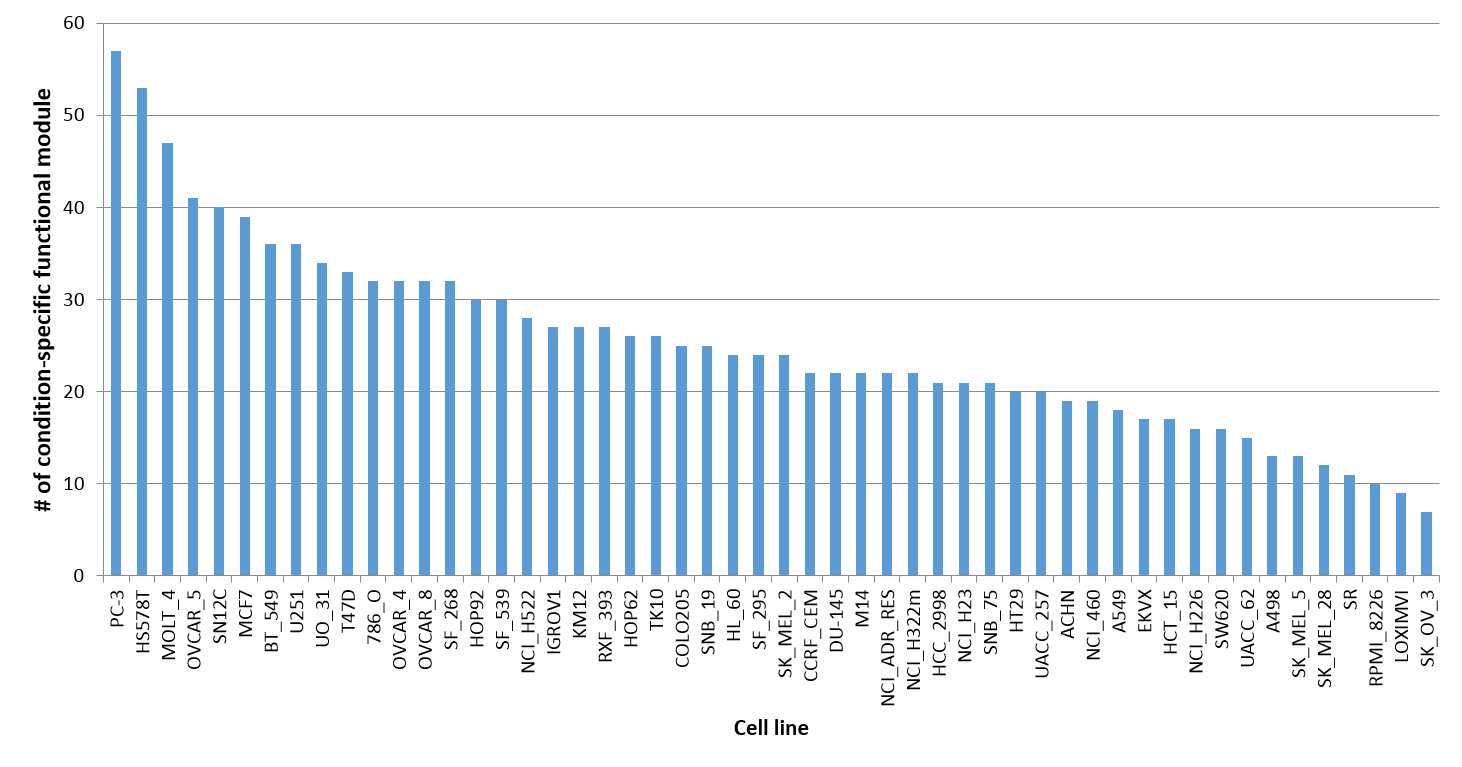

Supplement: Additional file 1: — Number of functional modules (TIF 161 kb) [file 12859_2016_1078_MOESM1_ESM.tif]
