# Supplementary material for: Context-specific functional module based drug efficacy prediction
Source: BMC Bioinformatics. 2016 Jul 28;17(Suppl 6):275. doi: 10.1186/s12859-016-1078-6 (PMC4965733; doi:10.1186/s12859-016-1078-6)
Supplement: Additional file 4: — GO terms of function vectors. Number of context-specific functional modules of cell lines (DOCX 18 kb) [file 12859_2016_1078_MOESM4_ESM.docx]

| Function | GO ID | GO description |
| --- | --- | --- |
| 1 | GO:0000377 | RNA splicing, via transesterification reactions with bulged adenosine as nucleophile |
|  | GO:0000375 | RNA splicing, via transesterification reactions |
|  | GO:0006139 | Nucleobase, nucleoside, nucleotide and nucleic acid metabolic process |
|  | GO:0006396 | RNA processing |
|  | GO:0000398 | Nuclear mRNA splicing, via spliceosome |
|  | GO:0016070 | RNA metabolic process |
|  | GO:0044260 | Cellular macromolecule metabolic process |
| 66 | GO:0010604 | Positive regulation of macromolecule metabolic process |
|  | GO:0010740 | Positive regulation of protein kinase cascade |
|  | GO:0008284 | Positive regulation of cell proliferation |
|  | GO:0007165 | Signal transduction |
|  | GO:0050865 | Regulation of cell activation |
|  | GO:0002684 | Positive regulation of immune system process |
|  | GO:0051251 | Positive regulation of lymphocyte activation |
|  | GO:0048522 | Positive regulation of cellular process |
|  | GO:0048518 | Positive regulation of biological process |
|  | GO:0031399 | Regulation of protein modification process |
|  | GO:0042127 | Regulation of cell proliferation |
|  | GO:0042531 | Positive regulation of tyrosine phosphorylation of STAT protein |
|  | GO:0002694 | Regulation of leukocyte activation |
|  | GO:0051704 | Multi-organism process |
|  | GO:0046425 | Regulation of JAK-STAT cascade |
|  | GO:0001932 | Regulation of protein amino acid phosphorylation |
|  | GO:0042327 | Positive regulation of phosphorylation |
|  | GO:0046427 | Positive regulation of JAK-STAT cascade |
|  | GO:0051247 | Positive regulation of protein metabolic process |
|  | GO:0045937 | Positive regulation of phosphate metabolic process |
|  | GO:0009967 | Positive regulation of Signal transduction |
|  | GO:0010647 | Positive regulation of cell communication |
|  | GO:0010628 | Positive regulation of gene expression |
|  | GO:0001934 | Positive regulation of protein amino acid phosphorylation |
|  | GO:0050864 | Regulation of B cell activation |
|  | GO:0032270 | Positive regulation of cellular protein metabolic process |
|  | GO:0019221 | Cytokine-mediated Signaling pathway |
|  | GO:0050731 | Positive regulation of peptidyl-tyrosine phosphorylation |
|  | GO:0050871 | Positive regulation of B cell activation |
|  | GO:0050867 | Positive regulation of cell activation |
|  | GO:0010627 | Regulation of protein kinase cascade |
|  | GO:0009893 | Positive regulation of metabolic process |
|  | GO:0002696 | Positive regulation of leukocyte activation |
|  | GO:0031401 | Positive regulation of protein modification process |
|  | GO:0010562 | Positive regulation of phosphorus metabolic process |
|  | GO:0051249 | Regulation of lymphocyte activation |
| 305 | GO:0002520 | Immune system development |
|  | GO:0002329 | Pre-B cell differentiation |
|  | GO:0030097 | Hemopoiesis |
|  | GO:0048534 | Hemopoietic or lymphoid organ development |
|  | GO:0002327 | Immature B cell differentiation |
| 472 | GO:0051056 | Regulation of small GTPase mediated signal transduction |
|  | GO:0050790 | Regulation of catalytic activity |
|  | GO:0043087 | Regulation of GTPase activity |
| 501 | GO:0009890 | Negative regulation of biosynthetic process |
|  | GO:0016481 | Negative regulation of transcription |
|  | GO:0007389 | Pattern specification process |
|  | GO:0009966 | Regulation of signal transduction |
|  | GO:0031327 | Negative regulation of cellular biosynthetic process |
|  | GO:0051172 | Negative regulation of nitrogen compound metabolic process |
|  | GO:0010629 | Negative regulation of gene expression |
|  | GO:0009892 | Negative regulation of metabolic process |
|  | GO:0003002 | Regionalization |
|  | GO:0031324 | Negative regulation of cellular metabolic process |
|  | GO:0010558 | Negative regulation of macromolecule biosynthetic process |
|  | GO:0045934 | Negative regulation of nucleobase, nucleoside, nucleotide and nucleic acid metabolic process |
|  | GO:0048729 | Tissue morphogenesis |
|  | GO:0035295 | Tube development |
|  | GO:0010605 | Negative regulation of macromolecule metabolic process |
|  | GO:0048598 | Embryonic morphogenesis |
| 502 | GO:0051173 | Positive regulation of nitrogen compound metabolic process |
|  | GO:0045935 | Positive regulation of nucleobase, nucleoside, nucleotide and nucleic acid metabolic process |
|  | GO:0051252 | Regulation of RNA metabolic process |
|  | GO:0045449 | Regulation of transcription |
|  | GO:0050789 | Regulation of biological process |
|  | GO:0051171 | Regulation of nitrogen compound metabolic process |
|  | GO:0031668 | Cellular response to extracellular stimulus |
|  | GO:0080090 | Regulation of primary metabolic process |
|  | GO:0031326 | Regulation of cellular biosynthetic process |
|  | GO:0031328 | Positive regulation of cellular biosynthetic process |
|  | GO:0031323 | Regulation of cellular metabolic process |
|  | GO:0048518 | Positive regulation of biological process |
|  | GO:0065007 | Biological regulation |
|  | GO:0009891 | Positive regulation of biosynthetic process |
|  | GO:0009889 | Regulation of biosynthetic process |
|  | GO:0006357 | Regulation of transcription from RNA polymerase II promoter |
|  | GO:0006355 | Regulation of transcription, DNA-dependent |
|  | GO:0050794 | Regulation of cellular process |
|  | GO:0010556 | Regulation of macromolecule biosynthetic process |
|  | GO:0019219 | Regulation of nucleobase, nucleoside, nucleotide and nucleic acid metabolic process |
|  | GO:0009991 | Response to extracellular stimulus |
|  | GO:0010468 | Regulation of gene expression |
|  | GO:0006952 | Defense response |
|  | GO:0060255 | Regulation of macromolecule metabolic process |
|  | GO:0010628 | Positive regulation of gene expression |
|  | GO:0009629 | Response to gravity |
|  | GO:0045941 | Positive regulation of transcription |
|  | GO:0009605 | Response to external stimulus |
|  | GO:0010557 | Positive regulation of biological process |
|  | GO:0048518 | Biological regulation |
|  | GO:0065007 | Positive regulation of biosynthetic process |
|  | GO:0009891 | Regulation of biosynthetic process |
|  | GO:0009889 | Regulation of transcription from RNA polymerase II promoter |
|  | GO:0006357 | Regulation of transcription, DNA-dependent |
|  | GO:0006355 | Regulation of cellular process |
|  | GO:0050794 | Positive regulation of macromolecule biosynthetic process |
| 503 | GO:0043526 | Neuroprotection |
| 504 | GO:0051173 | Positive regulation of nitrogen compound metabolic process |
|  | GO:0002376 | Immune system process |
|  | GO:0006139 | nucleobase, nucleoside, nucleotide and nucleic acid metabolic process |
|  | GO:0051252 | Regulation of RNA metabolic process |
|  | GO:0045449 | Regulation of transcription |
|  | GO:0030097 | Hemopoiesis |
|  | GO:0051171 | Regulation of nitrogen compound metabolic process |
|  | GO:0045893 | Positive regulation of transcription, DNA-dependent |
|  | GO:0080090 | Regulation of primary metabolic process |
|  | GO:0031326 | Regulation of cellular biosynthetic process |
|  | GO:0031328 | Positive regulation of cellular biosynthetic process |
|  | GO:0031323 | Regulation of cellular metabolic process |
|  | GO:0051254 | Positive regulation of RNA metabolic process |
|  | GO:0009891 | Positive regulation of biosynthetic process |
|  | GO:0009889 | Regulation of biosynthetic process |
|  | GO:0006357 | Regulation of transcription from RNA polymerase II promoter |
|  | GO:0006355 | Regulation of transcription, DNA-dependent |
|  | GO:0010556 | Regulation of macromolecule biosynthetic process |
|  | GO:0019219 | Regulation of nucleobase, nucleoside, nucleotide and nucleic acid metabolic process |
|  | GO:0010468 | Regulation of gene expression |
|  | GO:0060255 | Regulation of macromolecule metabolic process |
|  | GO:0006350 | Transcription |
|  | GO:0010628 | Positive regulation of gene expression |
|  | GO:0045941 | Positive regulation of transcription |
|  | GO:0045944 | Positive regulation of transcription from RNA polymerase II promoter |
|  | GO:0010557 | Positive regulation of macromolecule biosynthetic process |
|  | GO:0045935 | Positive regulation of nucleobase, nucleoside, nucleotide and nucleic acid metabolic process |
| 506 | GO:0022613 | ribonucleoprotein complex biogenesis" |
|  | GO:0050657 | nucleic acid transport |
|  | GO:0000956 | nuclear-transcribed mRNA catabolic process |
|  | GO:0006402 | mRNA catabolic process |
|  | GO:0000184 | nuclear-transcribed mRNA catabolic process, nonsense-mediated decay |
|  | GO:0006405 | RNA export from nucleus |
|  | GO:0044085 | cellular component biogenesis |
|  | GO:0015931 | nucleobase, nucleoside, nucleotide and nucleic acid transport |
|  | GO:0050658 | RNA transport |
|  | GO:0006401 | RNA catabolic process |
|  | GO:0006406 | mRNA export from nucleus |
|  | GO:0006403 | RNA localization |
|  | GO:0051236 | Establishment of RNA localization |
|  | GO:0051028 | mRNA transport |
|  | GO:0042254 | Ribosome biogenesis |
